# Abnormalities of Frontal-Parietal Resting-State Functional Connectivity Are Related to Disease Activity in Patients with Systemic Lupus Erythematosus

**DOI:** 10.1371/journal.pone.0074530

**Published:** 2013-09-12

**Authors:** Jingming Hou, Yun Lin, Wei Zhang, Lingheng Song, Wenjing Wu, Jian Wang, Daiquan Zhou, Qinghua Zou, Yongfei Fang, Mei He, Haitao Li

**Affiliations:** 1 Department of Radiology, Southwest Hospital, Third Military Medical University, Chongqing, China; 2 Department of Rheumatology, Southwest Hospital, Third Military Medical University, Chongqing, China; 3 Department of Clinical Psychology, Third Military Medical University, Chongqing, China; College of Mechatronics and Automation, National University of Defense Technology, China

## Abstract

Cerebral involvement is common in patients with systemic Lupus erythematosus (SLE) and is characterized by multiple clinical presentations, including cognitive disorders, headaches, and syncope. Several neuroimaging studies have demonstrated cerebral dysfunction during different tasks among SLE patients; however, there have been few studies designed to characterize network alterations or to identify clinical markers capable of reflecting the cerebral involvement in SLE patients. This study was designed to characterize the profile of the cerebral activation area and the functional connectivity of cognitive function in SLE patients by using a task-based and a resting state functional magnetic resonance imaging (fMRI) technique, and to determine whether or not any clinical biomarkers could serve as an indicator of cerebral involvement in this disease. The well-established cognitive function test (Paced Visual Serial Adding Test [PVSAT]) was used. Thirty SLE patients without neuropsychiatric symptoms and 25 age- and gender-matched healthy controls were examined using PVSAT task-based and resting state fMRI. Outside the scanner, the performance of patients and the healthy controls was similar. In the PVSAT task-based fMRI, patients presented significantly expanded areas of activation, and the activated areas exhibited significantly higher functional connectivity strength in patients in the resting state. A positive correlation existed between individual connectivity strength and disease activity scoring. No correlation with cerebral involvement existed for serum markers, such as C3, C4, and anti-dsDNA. Thus, our findings may shed new light on the pathologic mechanism underlying neuropsychiatric SLE, and suggests that disease activity may be a potential effective biomarker reflecting cerebral involvement in SLE.

## Introduction

Systemic lupus erythematosus (SLE) is a chronic autoimmune disease characterized by multisystem involvement in which neuropsychiatric manifestations are a common cause of significant morbidity, with an estimated prevalence as high as 76% [1]. Neurocognitive impairment (NCI) is rated as one of the most common and clinically challenging manifestations in patients with SLE, affecting as many as 59% of children and adolescents [1–3], which significantly decreases the quality of life [4]. NCI may be very subtle clinically and difficult to recognize, making the long-term consequence of NCI in SLE patients challenging.

Currently, formal neuropsychologic assessment is considered the standard for diagnosing cognitive dysfunction in SLE patients [5], but the pathophysiology underlying NCI remains unclear and continues to be an active focus of research. A number of studies using conventional MRI have shown brain gray and white matter abnormalities in SLE patients [6–9]; however, these findings failed to elucidate the pathophysiology underlying NCI in patients with SLE. It has been suggested that blood oxygen level-dependent functional magnetic resonance imaging (BOLD fMRI) is a promising approach to study human cognitive function, which utilizes deoxyhemoglobin as an endogenous contrast agent to identify areas of increased perfusion. Using fMRI, several studies have reported working memory [10], motor control [11], attention and language processing [12], and executive function [13] alterations in SLE patients; however, most of the results obtained in the above fMRI studies were performed with neuropsychiatric SLE (NPSLE) patients, while few studies were designed to characterize brain cognitive function alterations in lupus patients without obvious neuropsychiatric symptoms. Given the high prevalence and poor prognosis for NPSLE, it is meaningful to address whether or not brain dysfunction precedes the evolution of NPSLE in lupus patients without clinically obvious neuropsychiatric symptoms. This would lead to a better understanding of the pathogenesis underlying NPSLE, and provide potential direction for the development of early interventions.

The types of cognitive impairment which are most often reported in lupus patients without clinically obvious neuropsychiatric symptoms include executive function (working memory and planning) and attention function (delayed information processing and visual attention deficits) [14–16]. A variety of neuropsychological tests has been developed to investigate brain cognitive function. The Paced Visual Serial Addition Test (PVSAT) task is one of the most commonly used experimental paradigms in the evaluation of NCI [17]. Specifically, the PVSAT has been widely used to test sustained attention, working memory, and speed of information processing [17,18]. fMRI studies have been conducted with the PVSAT task in different clinical patients and in healthy controls [17,19,20], and have consistently shown activation of the fronto-parietal areas of the brain involved in attention processing and working memory. Thus, the PVSAT task appears to be particularly well-suited for studying the cognitive impairment involved in lupus patients without neuropsychiatric symptoms.

In recent years there has been increasing evidence that neural networks are disrupted in a number of neuropsychiatric diseases, such as Alzheimer’s disease [21], multiple sclerosis [22], schizophrenia [23], and depression [24]; however, no studies have determined whether or not lupus patients have abnormal neural networks. To explore the role of brain functional connectivity alterations in the development of cognitive impairment in SLE patients, we combined task-related fMRI with resting-state fMRI in the current study. Resting-state fMRI is a recently developed fMRI method which has been shown to be a promising approach to assess the functional connectivity of activation regions. Low-frequency (0.01–0.08 Hz) fluctuations of the BOLD signal in resting state fMRI are thought to be physiologically meaningful and reflect spontaneous neural activity in humans [25,26]. Such functional connectivity analysis is in widespread use by neuroimaging studies in various models [27–29].

It remains unclear whether or not the brain function alterations are associated with abnormal serum or clinical biomarkers in SLE patients. This issue is critical for identifying the etiopathogenesis of NPSLE. No studies have determined whether or not any clinical marker is capable of reflecting the cerebral involvement in SLE patients using fMRI. The purpose of the present study was as follows: 1) identify brain functional activation during a PVSAT task in SLE patients; 2) investigate functional connectivity of these activated areas in the resting state in SLE patients; and 3) determine whether or not serum or clinical biomarkers, such as the SLE disease severity index (SLEDAI), C3, C4, and anti-dsDNA, are correlated with the cerebral involvement indicated by functional activation or resting state connectivity.

## Materials and Methods

### Participants

The Medical Ethics Committee of the Third Military Medical University (Chongqing, China) approved the current study and all participants gave written informed consent to participation according to the Declaration of Helsinki. A total of 30 right-handed female SLE patients (35.42±7.95 years of age; 11.63±3.23 years of education) and 25 healthy controls (33.00±8.68 years of age, 12.84±3.10 years of education) were recruited from the Department of Rheumatology of Southwest Hospital. The diagnosis of SLE was made by consensus of attending physicians using the American College of Rheumatology (ACR) SLE criteria [30] and ACR case definition [5]. The Systemic Lupus Erythematosus Disease Activity Index (SLEDAI) was used to evaluate disease activity of SLE [31]. Twenty-one patients in this study had high levels of disease activity, as indicated by SLEDAI scores >4, while the other 9 patients had SLEDAI scores ≤4. Serum levels of complement components (C3, C4) and anti-dsDNA antibodies were measured in the hospital clinical laboratory (Table 1). At the time of study enrollment, all of the lupus patients were taking prednisone, with a mean±SD dose of 18.9±15.6 mg/day. At the time of enrollment, some lupus patients were also prescribed immunosuppressants (18 patients), non-steroidal anti-inflammatory drugs (NSAIDs; 8 patients), and antihypertensive medications (3 patients).

**Table 1:** Demographics, clinical characteristics, and PVSAT-100 performance of SLE patients and healthy controls.

Characteristics	SLE patients (n=30)	Controls (n=25)	*P*-value
Mean age (years)	35.4±8.0	33.0±8.7	0.285
Education (years)	11.6±3.2	12.8±3.1	0.166
Height (cm)	158.1±6.3	159.2±7.3	0.36
Weight (kg)	55.3±7.4	55.0±6.1	0.879
Disease duration (years)	5.0±4.3	_	_
SLEDAI score	6.8±4.0	_	_
C3 (mg/dL,normal range 85–193)	73.5±21.6	_	_
C4 (mg/dL, normal range 12-40)	10.3±7.9	_	_
Anti-dsDNA (IU/mL, normal range 0-200)	279.3±260.6	_	_
Prednisolone (mg/d)	18.9±15.6	_	_
PVSAT-100 performance (correct rate%)	93.6±4.4	92.8±4.1	0.455

Data are expressed as mean ± SD, SD: standard deviation; SLE: Systemic lupus erythematosus; SLEDAI: Systemic Lupus Erythematosus Disease ActivityIndex; Anti-dsDNA: anti-double-stranded DNA; PVSAT: Paced visual serial addition test.

The healthy controls recruited from the local community by poster advertisements were matched with the SLE patients for age, height, handedness, weight, and duration of education. All of the enrolled subjects were right-handed, as determined by the Edinburgh Handedness Inventory [32]. Before scanning, each subject underwent an off-scanner PVSAT-100 test in a separate and quiet room.

The following exclusion criteria were applied to all subjects: organic brain disorders; alcohol or drug abuse; pregnancy; or any physical illness, such as hepatitis, brain tumors, or epilepsy. The brain MR images (*T*_1_- and *T*_2_-weighted images) were inspected by two experienced neuroradiologists to exclude any gross structural abnormalities of the brain.

### MRI Acquisition

MRI was performed on a 3.0 T MR system (TIM Trio; Siemens, Erlangen, Germany). Each subject had a brain MRI examination with structural, resting state fMRI, and task-based fMRI sequences; the series covered the entire brain and was acquired without gaps between slices. The high-resolution structural *T*_1_-weighted image was scanned using a volumetric 3D magnetized prepared rapid gradient echo (MP-RAGE) sequence, as follows: repetition time/echo time (*T*_R_/*T*_E_), 1900 ms/3.45 ms; flip angle, 15°; slice thickness, 1 mm; matrix, 256 × 256; FOV, 256 mm × 256 mm; isotropic voxel, 1 mm ×1 mm × 1 mm; and total scan time, 270 s. The resting state fMRI was acquired by a gradient-echo echo-planar imaging (EPI) sequence, as follows: *T*_R_/*T*_E_, 2000 ms/30 ms; flip angle, 90°; slice thickness, 4 mm; matrix, 64 × 64; FOV, 256 mm × 256 mm; and isotropic voxel, 4mm × 4 mm × 4 mm. Each brain volume consisted of 36 axial slices and each functional run contained 210 image volumes preceded by five dummy volumes, resulting in a total scan time of 430 s. Participants were instructed not to focus their thoughts on any particular topic and to keep their eyes closed during resting-state fMRI acquisition. The task-based fMRI series were collected by an axial EPI sequence, as follows: *T*_R_/*T*_E_, 3000 ms/30 ms; flip angle, 90°; slice thickness, 4 mm; matrix, 64 × 64; FOV, 256 mm × 256 mm; and isotropic voxel, 4 mm ×4 mm × 4 mm. Each brain volume comprised 36 axial slices and each functional scanning session consisted of three runs, and each run contained 55 image volumes preceded by five dummy volumes, resulting in a total scan time of 540 s.

### fMRI PVSAT Task

**Figure 1:**
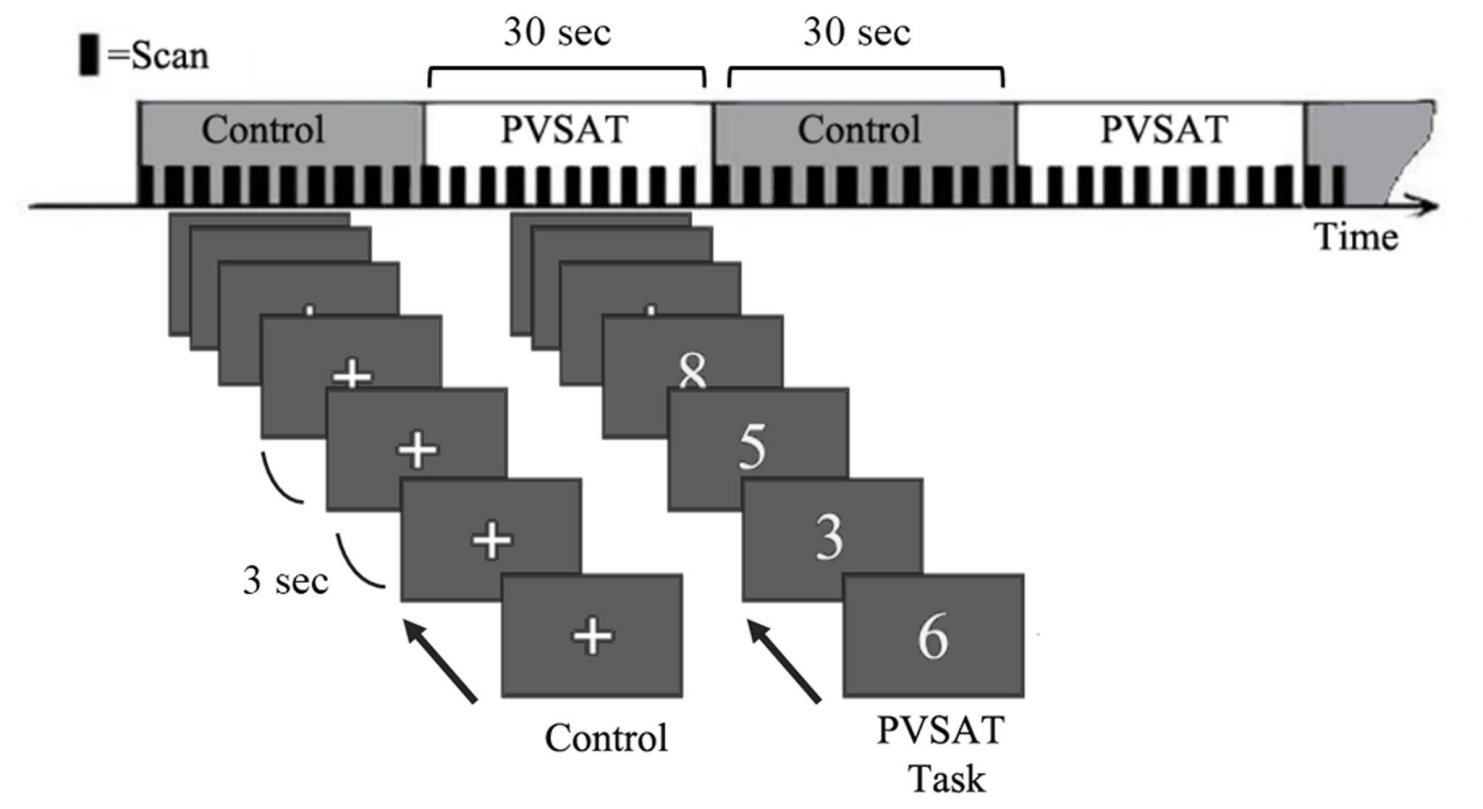
PVSAT task during fMRI. During the PVSAT phase, a random series of numbers (range, 1–9) was presented one at a time and the subject was instructed to consecutively add pairs of numbers. The baseline visual task consisted of the presentation of a fixed cross. In both tasks, each stimulus was presented for 1 s, followed by a blank screen for 2 s.

We used a paradigm similar to that applied successfully in a PVSAT fMRI study of patients with multiple sclerosis [33]. The trial consisted of three fMRI runs, each run consisting of 180 s (total = 540 s). Within each run, the subject completed the PVSAT (active task) or a baseline visual task (control) according to a boxcar paradigm with three 30 s active task periods alternating with three 30 s control periods (Figure 1). The visual stimulus was projected onto a screen inside the MR room, and a mirror was placed on the head coil at a 45° angle to the screen and the subject’s line of sight. During the PVSAT phase, a random series of numbers (range, 1–9) was presented one at a time and the subject was instructed to consecutively add pairs of numbers, i.e., each number was added to the number that immediately preceded it. The baseline visual task consisted of the presentation of a fixed cross. In both tasks, each stimulus was presented for 1 s, followed by a blank screen for 2 s to ensure that the number was stored, and retrieved from working memory for further processing. Before MRI scanning, all subjects were trained in the experimental protocol outside the scanner to ensure that they had understood the instructions correctly and could perform the task during scanning, and while in the scanner, the subjects were instructed to calculate silently.

### Data Pre-processing

Functional image preprocessing and statistical analysis were done with SPM8 software (Statistical Parametric Mapping; http://www.fil.ion.ucl.ac.uk/spm/). To avoid manipulation error confounders and to standardize the process, we used the batch-processing tool Data Processing Assistant for Resting-State fMRI (DPARSF; http://www.restfmri.net) [34]. For both the resting state and task-based fMRI data of each subject, the first five volumes of functional images were discarded to allow for steady-state magnetization and stabilization of participant status. EPI images were slice-time corrected to the middle slice acquired in time, and realigned and re-sliced to correct for head motion with a mean volume created. Structural images were co-registered with the mean volume of functional images and subsequently segmented by an inbuilt unified segmentation routine in SPM8 [35]. The parameter created by segmentation was then applied to functional images with non-linear normalization to the Montreal Neurological Institute (MNI) template brain, and each voxel was re-sampled to isotropic 3 mm × 3 mm × 3 mm. Both the resting state and task-based fMRI images were smoothed using an 8-8-8 mm FWHM Gaussian kernel. The head motion of all participants during resting-state fMRI acquisition was observed, and data were discarded if the translation exceeded 2 mm or if rotation exceeded 2°.

### Task-based fMRI Analysis

The effect of the PVSAT task was estimated using a general linear model. The data for each subject were modeled using a boxcar model convolved with the hemodynamic response function. Estimated head movement parameters from realignment were included as covariates at this first level of analysis. Voxel values for task-versus-control contrast yielded a statistical parametric map of the *t* statistic, and were then normalized to *Z*-scores. A corresponding contrast image for each patient was also created for group analysis. Group analysis was performed using a fixed effects model to explore common activation within each group. A one-sample *t*-test was applied to the contrast images of the subjects in each group. Finally, we used a random effect model in performing between-group analyses to show the difference of the activation areas between SLE patients and healthy controls. A two-sample *t*-test was applied to determine areas that showed greater or weaker activation in the SLE patients compared with the healthy controls. We used a height threshold of *p*<0.05 FWE-corrected and an extent threshold of 30 voxels/cluster. The *Z* value of activation areas from an individual patient were extracted and tested for correlation with serum markers and clinical indices.

### Functional Connectivity Analysis

Functional connectivity was analyzed using the REST software package (http://www.restfmri.net) using a method based on regions of interest (ROIs) wise analysis [36,37]. Because task-based fMRI exhibited an alteration of activation in SLE patients, we tested whether or not the intrinsic connectivity of these areas had been affected. The ROIs were defined as the entire activated areas that resulted from task-based fMRI group analysis of the healthy controls. In this study, the ROIs were extracted and re-sampled into 3-mm cubic voxels using the REST software. The time series of each ROI was pre-processed as follows: six head motion parameters, the averaged signals from cerebrospinal fluid, white matter, and the global brain signal were removed from the data through linear regression; the time series was band filtered (0.01–0.08 Hz) and linear-trends were removed to reduce the effects of low-frequency drift and high frequency noise; the averaged time series for all voxels within the ROIs were extracted and tested for synchronization between times series within each ROI to calculate an *r*-value representing connectivity strength. The functional connectivity strength between the two groups was compared using Student’s *t*-test for independent samples (statistical significance was set at a *p*≤0.05), and tested for linear correlation between the individual connectivity strength of patients and serum biomarkers, such as C3, C4, and anti-dsDNA, and clinical indices of disease activity, disease duration, age, education, and daily glucocorticoid dose using SPSS 18.0 software (SPSS, Inc., Chicago, IL, USA).

## Results

### PVSAT performance

We acquired whole-brain fMRI in PVSAT tasks and the resting state for 30 female lupus patients and 25 healthy controls. Before scanning, an off-scanner PVSAT-100 test was administered in a separate and quiet room. During the PVSAT-100 test, SLE patients and the controls gave a similar number of correct answers (93.62±4.35 and 92.76±4.07, respectively). There was no significant correlation between PVSAT scores and duration of formal education for either group (*p*>0.05). Table 1 summarizes the demographic data, clinical characteristics, and PVSAT-100 performance data of the study subjects.

### Task-based fMRI activations

**Figure 2:**
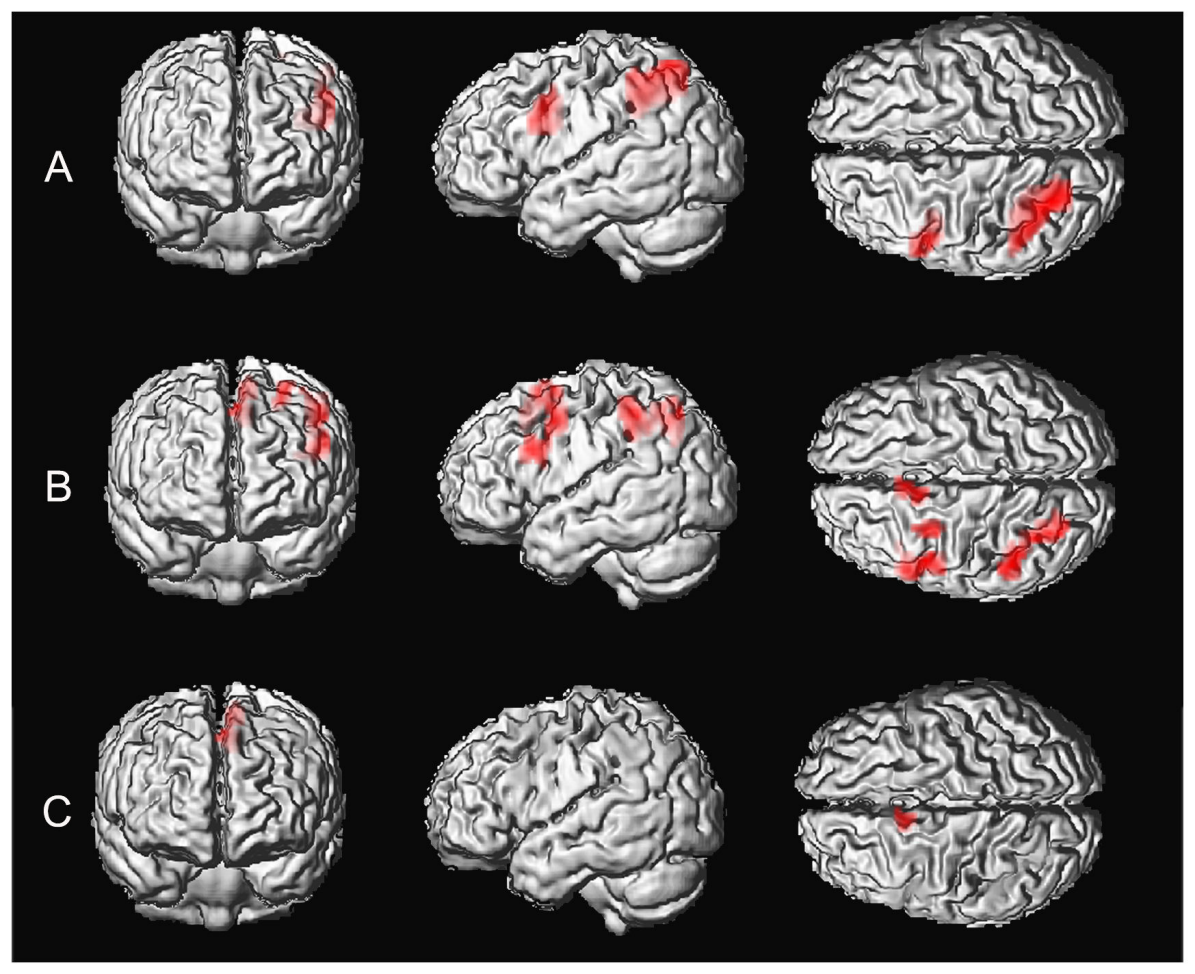
PVSAT baseline task activations for control subjects (A) and SLE patients (B). All of the reported activation clusters survived a FEW-corrected *p*<0.05 at the voxel level and an extent threshold of 30 voxels. In control subjects, the activations were situated in the left superior and inferior parietal lobes, and the left inferior frontal gyrus. For SLE patients, significantly extended activation was found in the left hemisphere in the superior and inferior parietal lobes, the supplementary motor area, and the middle and inferior frontal gyrus. SLE patients (compared to controls) demonstrated increased activation mainly in the left supplementary motor area (C).

fMRI scans were collected while subjects performed a PVSAT paradigm. Brain activation maps during the PVSAT task in controls and SLE patients are shown in Figure 2, and activation peak coordinates are summarized in Table 2. All of the reported activation survived an FWE-corrected *p*<0.05 threshold at the voxel level and an extent threshold of 30 voxels. In the controls, the activation was situated in the left superior and inferior parietal lobes and the left inferior frontal gyrus. In the SLE patients, significantly extended activation was found in the left hemisphere in the superior and inferior parietal lobes, the supplementary motor area, the inferior frontal gyrus, and the middle frontal gyrus. Compared to healthy controls, SLE patients showed greater activation mainly in the left supplementary motor area. There was no area that exhibited decreased activation in the SLE patients than in the healthy controls (FWE corrected, *p*<0.05).

**Table 2:** Activated regions during PVSAT task in healthy controls and SLE patients.

Brain region	BA	Side		Cluster size	MNI coordinate			P value (FWE)	T score	Z score
					x	y	z			
**Activated regions in normal controls**										
Superior parietal lobule	7		L	133	-27	-60	54	<0.001	12.79	6.96
Inferior parietal lobule	40		L	190	-29	-61	39	<0.001	10.13	6.13
Inferior frontal gyrus	9		L	113	-48	6	36	<0.001	9.43	5.94
**Activated regions in SLE patients**										
Supplementary motor area	32		L	88	-6	18	45	<0.001	9.56	6.00
Middle frontal gyrus	6		L	107	-45	3	42	<0.001	9.51	5.98
Inferior Parietal lobule	40		L	185	-30	-66	42	<0.001	9.50	5.98
Inferior frontal gyrus	9		L	98.	-30	3	60	<0.001	9.59	6.01
**SLE patients>normal controls**										
Supplementary motor area	32		L	63	-8	20	43	<0.001	9.36	5.92
**SLE patients<normal controls** None										

BA: broadmann area; L: left (*P*<0.05, corrected with FWE). The anatomical localization was implemented by using xjview software.

### Functional connectivity

**Figure 3:**
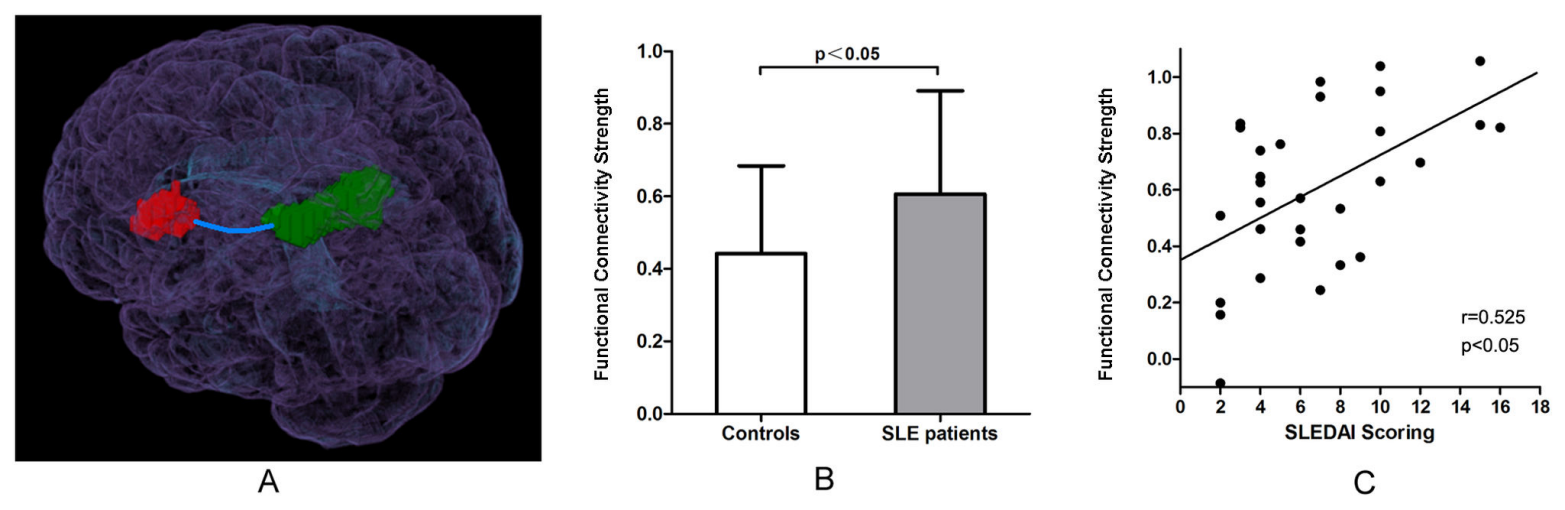
Results of functional connectivity strength analysis. Activation areas of the PVSAT task in normal controls were selected as ROIs to test for connectivity strength (A). Compared to the controls, SLE patients exhibited significantly intensified (*p*<0.05) connectivity strength (B); nevertheless, the SLE disease activity index (SLEDAI) score was positively correlated (*r*=0.525, *p*<0.05) with individual connectivity strength in patients (C).

We utilized functional connectivity analysis based on fMRI scans acquired during the resting state to further investigate alterations of the cerebral network of cognitive function in SLE patients. Brain areas activated in healthy controls during the PVSAT task were selected as ROIs to test for the strength of connectivity between patients and controls (Figure 3A). Compared to the controls, the SLE patients exhibited a significantly higher magnitude (*p*<0.05) of connectivity strength between areas activated during the PVSAT task (Figure 3B), indicating hyperactivated synchronization between these areas.

### Correlation analysis

The correlation analysis was performed between the functional connectivity strength or *Z*-scores of activated areas in SLE patients during the PVSAT task-based fMRI and serum biomarkers, such as C3, C4, and anti-dsDNA, and clinical indices of disease duration, SLEDAI score, age, duration of education, and daily glucocorticoid dose. We found a significant positive correlation between connectivity strength and SLEDAI score (*r*=0.525, *p*<0.05), suggesting that such magnified connectivity strength is modulated by the disease activity of an individual patient (Figure 3C). Furthermore, no other correlation for the functional connectivity strength or *Z*-scores was observed for the serum biomarkers and clinical indices.

## Discussion

The present study characterized the profile of the cerebral activation area and the functional connectivity of cognitive function in SLE patients by using a task-based and resting state fMRI technique. Furthermore, we also explored whether or not a clinical biomarker could serve as an indicator of cerebral involvement in patients with SLE. In the PVSAT-related fMRI, patients presented significantly expanded areas of activation, and the activated areas exhibited significantly higher functional connectivity strength in patients during the resting state. A positive correlation was found between individual connectivity strength and SLEDAI score. No correlation with cerebral involvement was found for serum markers, such as C3, C4, and anti-dsDNA.

NCI, which negatively impacts the social functioning of SLE patients, is a common and critical symptom in patients without pre-existing major neuropsychiatric manifestations [38]. Results from earlier fMRI studies in patients with SLE using various tasks have demonstrated increased cerebral activation compared to healthy controls [10–12]. The phenomenon revealed by the current and earlier research indicating that SLE patients with possible cognitive impairment have higher activity in those areas on fMRI might be counterintuitive; however, a similar result was observed under other conditions in which cognitive impairment is a feature, including multiple sclerosis [39,40]. Different authors have proposed that such changes in activation patterns represent a plastic response to disease-related cerebral damage, and thus an attempt to compensate for the deficit due to brain injury. In the current study significantly extended activations were noted in lupus patients during the PVSAT-task fMRI, which indicated that lupus patients utilize broader cerebral areas than the controls to accomplish the PVSAT task. Interestingly, there was no difference between groups in PVSAT-100 performance accuracy, implying that these changes in activation patterns could represent a compensatory mechanism, allowing lupus patients to achieve the same level of cognitive function as healthy controls.

The most striking findings of the present study include the abnormalities of the frontal-parietal restingstate functional connectivity related to disease activity in lupus patients without obvious neuropsychiatric symptoms. We found increased functional connectivity in the frontal-parietal cortex during the resting state was positively correlated with the disease activity in lupus patients, which is consistent with the findings of previous structural studies (i.e., disease activity can influence brain structure in lupus patients) [41,42]. The positive correlation which existed between frontal-parietal functional connectivity strength and SLEDAI scoring supports the proposal that the disease state of SLE can impact on the cerebral function of lupus patients, even in the early stages. Previous structural and functional MRI studies also showed that abnormal brain structural or functional changes may occur before the appearance of any obvious central nervous system symptoms and conventional imaging signs [13,41–44]. These results indicate that greater attention must be paid to the involvement of the central nervous system in the early stages of SLE. We previously also demonstrated that a significant alteration of brain function revealed by resting state fMRI can be found in lupus patients without obvious neuropsychiatric symptoms [45]. As most of those altered areas were in the default mode network (DMN) [46,47], we speculated that areas of a specific performance network could be damaged. Results from the current study provided direct evidence that despite hyperactivity of areas during task performance in SLE patients, the abnormal functional connections of frontal-parietal regions presented also in the resting state. These findings provide direct evidence that significant alteration of brain function, resembling that observed in SLE patients with neuropsychiatric disorders, is already present in SLE patients without obvious neuropsychiatric complications, highlighting the need for early intervention and maintenance of patients in the early stages of SLE. Several experiments have tested whether or not a serum or neurologic marker could serve as a clinical index for cerebral involvement of SLE [48–51]; however, the results were inconsistent, partly because the criterion for diagnosing NPSLE was dependent on subjective standards. As a more objective tool, increasing numbers of studies have used fMRI as an investigative tool for evaluation of cerebral status [52–55]. As a newly introduced technique, resting state fMRI has been welcomed for its less artificial interference during scanning, leading to an experimental pattern that is easier for patients to understand. This advantage is particularly beneficial for studies of patients with impaired neurologic or psychological functions. This is the first neuroimaging study combined with task-based and resting state fMRI designed to determine whether or not a clinical biomarker could serve as an indicator of cerebral involvement in patients with SLE. Before the study, we expected that several serum or clinical markers would be correlated with cerebral alterations reflected by fMRI; however, none of the serum markers tested was found to correspond to the fMRI results. Our results indicated that dysfunction of the brain network is only correlated significantly with SLE disease activity.

In this study, we combined task-based and resting state fMRI and showed that altered cerebral functions were detected using both methods. Nevertheless, the reliability of such techniques in SLE patients should be further validated by future experiments. Of note, in the last few years multivariate methods have provided more objective neuroimaging-based biomarkers in neuropsychiatric disorders. By using these multivariate methods, important and interesting results have been reported by analyzing fMRI data [56–59]. For example, Zeng et al. [58] demonstrated that multivariate pattern analysis (MVPA) methods can identify major depressive individuals from healthy controls based on resting-state fMRI data with 94.3% accuracy. Using low-dimensional embedding of fMRI, Shen et al. [59] also discriminated schizophrenic patients from healthy controls with excellent accuracy and sensitivity. Future investigations in lupus patients are needed using the above multivariate methods as an objective neuroimaging-based biomarker for more reliable clinical evaluation and diagnosis.

The current study had several limitations. First, we failed to enroll more clinical and neurologic markers due to a limited budget. fMRI technology is mostly restricted to the laboratory rather than the clinical setting. The technology will prove to be good value if a cost-effective and clinical or neurologic marker is correlated with neuropsychological changes in such patients in the early stages of the disease. Second, it was not possible to identify a sufficient number of SLE patients with active CNS involvement to serve as positive reference controls. This was, in part, because of the diversity of neuropsychiatric symptoms displayed by SLE with overt central nervous system involvement, such as headache, cognitive dysfunction, affective disorders, and confusional states, making it difficult to accumulate a group of NPSLE patients with comparable neuropsychological presentations. In addition, some patients with clinically overt neuropsychiatric lupus might have difficulty in understanding and performing the PVSAT test. Thus, we decided to limit the patient groups to a cohort of SLE patients without clinically obvious neuropsychiatric symptoms to have a consistent clinical neuropsychological state among patients. Third, a methodologic issue that needs to be considered refers to the fact that the data in our analyses are based on ROIs approach. The grounds for choosing the PVSAT-based fMRI activated areas as seed regions seems solid given the importance of these regions for cognitive function; however, this approach inevitably yields arbitrary results. A way to overcome this might have been the use of multivariate pattern analysis, which has been demonstrated to be a more effective approach with excellent classification accuracy and sensitivity to explore potential biomarkers for neuropsychiatric disorders [58].

## Conclusion

Cognitive function is an indispensable part of human life, and patients with NPSLE often suffer from related difficulties. By combining task-based and resting state fMRI, we were able to extend our understanding of this illness in three important ways. First, our results showed activated cerebral involvement in a specific task brain network in SLE patients with intact performance outside the scanner, supporting the use of resting state fMRI along with traditional task-based techniques for revealing the alteration of a given task-specific network in the disease. Second, the serum markers in current use might be insufficient for revealing cerebral involvement of this disease. Third, the positive correlation between SLEDAI scoring and functional connectivity strength of brain areas supports the proposal that the general disease state of SLE has an impact on cerebral function of SLE patients, even in the early stages, highlighting the need for early intervention and maintenance of patients in the early stages of SLE.
